# CT scan chest findings in symptomatic COVID-19 patients: a reliable alternative for diagnosis

**DOI:** 10.4314/gmj.v54i4s.14

**Published:** 2020-12

**Authors:** Benjamin D Sarkodie, Yaw B Mensah

**Affiliations:** Department of Radiology, University of Ghana Medical School, Korle Bu, Accra

**Keywords:** Computed Tomography, COVID-19, Coronavirus disease 2019, RT-PCR: Reverse Transcription Polymerase Chain Reaction

## Abstract

Computed Tomography (CT) scan of the chest plays an important role in the diagnosis and management of Coronavirus disease 2019 (COVID-19), the disease caused by the novel coronavirus SARS-CoV-2. COVID-19 pneumonia shows typical CT Scan features which can aid diagnoses and therefore help in the early detection and isolation of infected patients. CT scanners are readily available in many parts of Ghana. It is able to show findings typical for COVID-19 infection of the chest, even in instances where Reverse Transcription Polymerase Chain Reaction (RTPCR) misses the diagnosis. Little is known about the diagnostic potential of chest CT scan and COVID-19 among physicians even though CT scan offers a high diagnostic accuracy.

## Introduction

The novel coronavirus disease 2019(COVID-19) is an infectious disease caused by the severe acute respiratory syndrome coronavirus2 (SARS-CoV-2).^1^ SARS-CoV-2 belongs to the coronavirus family responsible for respiratory complications.

The disease was reported in Wuhan, China, in December 2019 before spreading to many parts of the world by February, 2020.^2^ The World Health Organisation (WHO) confirmed reports of person-to-person spread and subsequently declared the disease a pandemic on 11^th^ March, 2020.^3^, COVID-19 has rapidly spread to over 210 countries and territories, with over 17.4 million cases and 675,584,000 deaths reported globally as of August 31^st^, 2020.^4^ The Ghana Health Service as of 30^th^ July 2020, had documented 35,142 confirmed cases with 175 deaths in Ghana.^5^

Fever, cough and difficulty in breathing are some of the common COVID-19 symptoms.^6^,^7^ Like all epidemics, the key strategies to prevent widespread transmission is to test/diagnose, isolate and treat individuals with suspected or confirmed COVID-19.^8^

### PCR testing issues and why CT scan of the chest

The gold standard for testing for the novel coronavirus is the Reverse Transcription Polymerase Chain Reaction (RT-PCR) method.^8^,^9^,^10^

RT-PCR, or PCR swab has a specificity 99% with a sensitivity of 50–80% thus potentially up to 50% cases of COVID-19 could be missed by PCR.

Due to the highly transmissible nature of COVID-19, the number of individuals who require testing is typically very high and potentially overwhelming for healthcare system. Problems with testing efficiency, reliability and delays with results availability have previously been reported^9^ with obvious implications for disease control and transmission prevention. Test results in centres in Ghana have been received after 2–4 weeks thus defeating the purpose of early diagnosis. In order to address this pitfall a lot of clinics in China at the peak of the COVID-19 infection did both PCR and chest CT for suspected cases.^11^

Studies in China showed that chest CT scan was able to pick some cases of COVID-19 pneumonia which were missed by the swab test.^12^ Many other studies have showed that CT scan of the chest is very reliable in the diagnoses of COVID-19 symptomatic patients. Several studies have reported that chest CT has a sensitivity of 80–90% and a specificity of 82.8–96% for detecting lung lesions in patients with COVID-19.^13^,^14^

The guidelines for Diagnosis and Treatment of Pneumonitis Caused by 2019-nCoV (trial sixth version) published by the Chinese Health Authority recommended chest CT as an effective method to screen suspicious cases.

The addition of chest CT for diagnosis of COVID-19 resulted in several thousands of clinically diagnosed cases in China which played an important role in controlling the epidemic in China.^15^ A policy direction that will encourage the utilization of CT scan as a complementary or alternative tool in the diagnosis of COVID 19 will help consolidate national and global efforts at controlling the virus.

Beyond this sensitivity, CT also offers the advantage of being able to interpret findings immediately hence once COVID-19 infection is suspected, patients can be isolated and treated early to reduce risk of spreading the infection. As there is still no approved rapid swab testing in Ghana at the time of this study, relying solely on PCR testing can potentially slow progress to tackle this virus. We need to use CT scan of the chest as a reliable complement to PCR testing. Countries like China and Singapore that have achieved a significant level of control have deployed wide use of CT scan of the chest.

### CT Scan /PCR Discordance

Between March and June 2020, we picked up 43 cases of COVID-19 chest infections on chest CT scans in a private clinic in Accra. Forty of these patients were subsequently confirmed by PCR during the first testing (40/43 Concordance). Of the remaining 3, a second PCR testing done confirmed 2 of them as positive while the third patient remained negative. We insisted on retesting because the imaging findings were typical for COVID-19 according to previously published imaging findings^16^,^17^,^18^,^19^ including bilateral, peripheral and subpleural ground glass opacities. RT-PCR test for 2019-nCoV may be falsely negative due to laboratory error or insufficient viral material in the specimen.^20^

### Case presentation of the three cases who initially tested negative

**Case 1:**
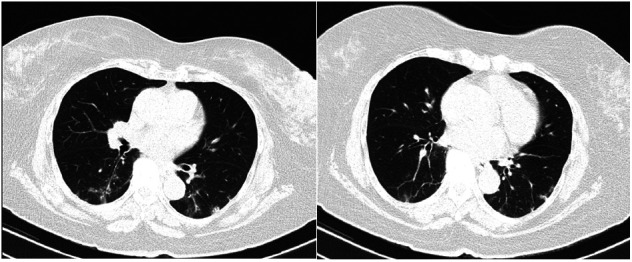
Seventy-seven-year-old female with a 5-day history of cough with fever and headaches. CT scan of the chest showing bilateral subpleural mixed ground-glass opacities GGO and consolidation with traction bronchiectasis (Negative initial PCR testing. Positive repeat PCR within 3 days)

**Case 2:**
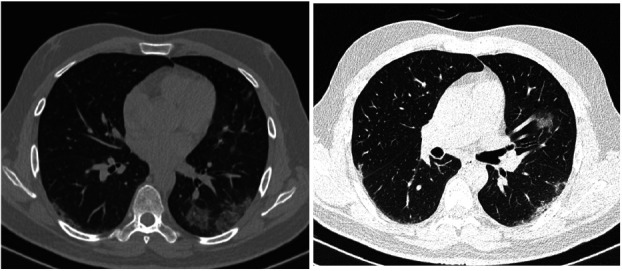
39-year-old male with history of vomiting and abdominal pain. No cough or fever. CT scan of the abdomen found incidental ground glass opacities at the lung bases for which a subsequent dedicated chest CT scan was requested showing bilateral and subpleural ground-glass opacities (Negative initial PCR testing. Positive repeat PCR within 3 days)

**Case 3:**
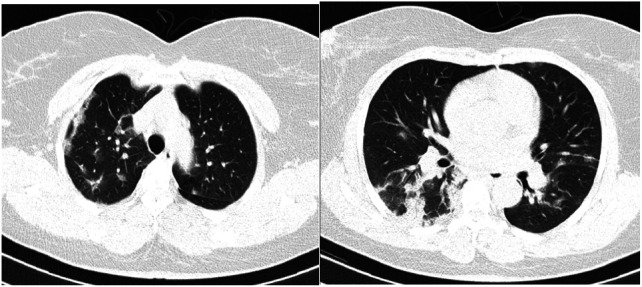
50 year old male with three-day history of cough and fever. CT scan of the chest showing bilateral, peripheral and subpleural consolidation (Negative initial PCR testing. Negative repeat PCR within 3 days)

In Ghana, a there are currently over 35 CT scanners dotted across the country (according to a document obtained from the Ghana's Nuclear Regulatory Agency in 2008) compared to the only three centres with PCR testing capabilities. Using the CT scan for triaging COVID-19 cases is certainly worth considering in patients who are symptomatic.

There are disadvantages associated with the use of the CT scan for triaging COVID-19 patients like radiation exposure and the higher cost compared to PCR but the benefits of controlling the virus far out weights these downsides. Also, the current PCR laboratory testing is time-consuming, and often plagued by shortage of supply test kits and may not meet the needs of the growing infected population. RT-PCR testing for 2019-nCoV may be falsely negative due to laboratory error or insufficient viral material in the specimen.^20^

## Conclusion

CT scan of the chest has been shown to have a high sensitivity and specificity for identifying lung lesions in COVID-19 infection. With the limited access to PCR testing and associated delay in receiving results the use of CT scan of the chest in triaging should be encouraged. In China and Korea where the virus has been largely put under control there is a widespread of CT scan of the chest to aid in diagnosis.
